# Food-specific IgG-based elimination diet decreased IL-6, TNF-α, and CGRP and improved symptoms in adults with migraine

**DOI:** 10.3389/fnut.2025.1720389

**Published:** 2025-12-15

**Authors:** Zhiming Zhao, Meimei Yang, Fujun Wan, Baoli Ning, Tao Song, Jun Fu, Liming Zhang

**Affiliations:** 1 Health Center of Screening and Prevention of Diseases, The First Affiliated Hospital of Harbin Medical University, Harbin, Heilongjiang, China; 2 Department of Neurology, The Fourth Affiliated Hospital of Harbin Medical University, Harbin, Heilongjiang, China; 3 Department of Neurology, The First Affiliated Hospital of Harbin Medical University, Harbin, Heilongjiang, China

**Keywords:** migraine, comorbidities, food-specific IgG, inflammatory cytokine, neuropeptide

## Abstract

**Background:**

Food-specific IgG antibodies have been proposed to be biomarkers to identify food that triggers an inflammation response. We aimed to evaluate the effect of a food-specific IgG-based elimination diet by assessing the changes in the symptoms of migraine and its comorbidities, inflammatory cytokines, neuropeptides, and neurotransmitters, and their correlation.

**Methods:**

This was a sham-controlled randomized trial. A total of 98 patients with migraine who had at least one positive food-specific IgG antibody were randomly assigned to either the true diet group (IgG-positive foods were excluded, *n* = 52) or the sham diet group (alternative IgG-negative foods were excluded, *n* = 46). At baseline and at the end of 12 weeks, seven questionnaires were administered regarding the symptoms of migraine, gastrointestinal symptoms, anxiety, depression, and sleep quality. Fourteen food-specific IgG antibodies, such as IL-6, IL-10, TNF-α, 5-HT, calcitonin gene-related peptide (CGRP), and vasoactive intestinal peptide (VIP), in serum were assessed using enzyme-linked immunosorbent assays.

**Results:**

At the end of 12 weeks, the true diet group had a larger reduction in questionnaires of migraine (except for MSQ), gastrointestinal symptoms, and poor sleeping, as well as food-specific positive IgG, IL-6, TNF-α, and CGRP in the serum. The difference in the change of “days with migraine in past 4 weeks” between the sham and true diet groups was significantly attenuated after adjusting for IL-6 and TNF-α.

**Conclusion:**

An IgG-positive food elimination diet improved migraine and its comorbidities and reduced IL-6, TNF-α, and CGRP, which might be associated with the alleviated systemic chronic inflammation and downregulation of the sensitivity of trigeminal nerve endings.

**Clinical trial registration:**

Chictr.org.cn, identifier ChiCTR2000039278.

## 1 Introduction

Migraine is one of the most common neurological disorders that can cause severe disability in patients. However, people have insufficient awareness and treatment of migraine (1). Comorbidity refers to the statistical association between two different diseases in the same individual by a ratio higher than expected by chance (2).

More and more evidence has suggested that depression, anxiety, sleep disorders, and gastrointestinal dysfunction are comorbidities of migraine (3–6). The symptoms of these comorbidities not only increase the difficulty of clinical diagnosis of migraine but also increase the complexity of treatment. Further research is needed to determine whether migraine and its comorbidities share a common pathogenesis and corresponding treatment methods.

Sensitivity to a particular food is one of the most common triggers of migraine (7). Immune response and inflammation play a crucial role in the origin and persistence of migraine (8). Although IgE explains immediate hypersensitivity, the delayed hypersensitivity is impossible to overlook. Non-IgE immunologic hypersensitivity to foods is common and can cause considerable morbidity (9). Consequently, a reliable specific marker is needed. The hypersensitivity reaction mediated by food-specific IgG antibodies may be explained by low-level absorption of food macromolecules from the gut, causing low-grade chronic inflammation (10). Some scholars have proposed that food-specific IgG antibodies may be a biomarker for migraines to identify foods associated with increased inflammation response *in vivo* (11, 12).

Neurogenic neuroinflammation has a wide role in migraine pathogenesis, with altered systemic immune responses occurring in migraine patients due to the release of pro-inflammatory cytokines, such as TNF-α and IL-6, both in ictal and interictal periods (13, 14). Meanwhile, IL-10 production also changes accordingly as an anti-inflammatory cytokine (15).

Upon activation by cytokines, mast cells release multiple neuropeptides and neurotransmitters, including 5-hydroxytryptamine (5-HT), calcitonin gene-related peptide (CGRP), and vasoactive intestinal peptide (VIP), which increase the excitability of endogenous intestinal neurons (regulating movement and secretion) and incoming exogenous neurons (also providing pain signals to the central nervous system) (16, 17).

Our previous cross-sectional study has shown that the effect of food-specific IgG antibodies on the severity of migraine and its comorbidities was antibody-quantity dependent and IgG-concentration dependent (18). Inflammatory cytokines, TNF-α and IL-6, might partially mediate the causal pathway between food-specific IgG antibodies and the symptoms (19). These results motivate us to conduct a prospective randomized trial to further investigate the impact of food-specific IgG-based elimination diet on migraine and its comorbidities, as well as the underlying mechanisms involving the inflammatory cytokines, neuropeptides, and neurotransmitters.

With this background, we examined the symptoms of migraine and its comorbidities, the 14 food-specific IgG antigens, IL-6, IL-10 and TNF-α, 5-HT, CGRP, and VIP, of adult migrant patients, and conducted a randomized controlled trial to compare the changes by two different food-specific IgG-based elimination diets. Then we further explored the correlation among them, which was also a key part of this study.

## 2 Materials and methods

### 2.1 Patients

Patients with migraine were consecutively recruited at the Department of Health Center of Screening and Prevention of Diseases, the First Affiliated Hospital of Harbin Medical University. International Classification of Headache Disorders, 3rd edition beta version (ICHD-3-beta), was employed to diagnose migraine by two neurologists (20). The inclusion criteria included the following: (1) 18–62 years old; (2) having at least one positive food-specific IgG; (3) having a diagnosis of migraine for more than 6 months and at least 2 episodes of migraine in the past 30 days. The exclusion criteria included: (1) headache caused by other diseases, which include, but not limited to, tumors, hypertension, stroke, unstable mental health disorders, peptic ulcer, anemia, infectious diseases, and autoimmune diseases; (2) current use of migraine-preventive treatments, food restriction intervention, immunosuppressive drugs, or antibiotics.

Our study proposed to recruit 90–100 patients with migraine and food-specific positive IgG with an estimated discontinuation rate of 10%. Instead of using power calculation, this sample size was determined based on the feasibility and practical ability to achieve the study’s purpose. Nevertheless, the sample size of the present study was not smaller than that of similar, previous studies on food IgG-based elimination diet (10, 21–24).

### 2.2 Study design

**Figure 1:**
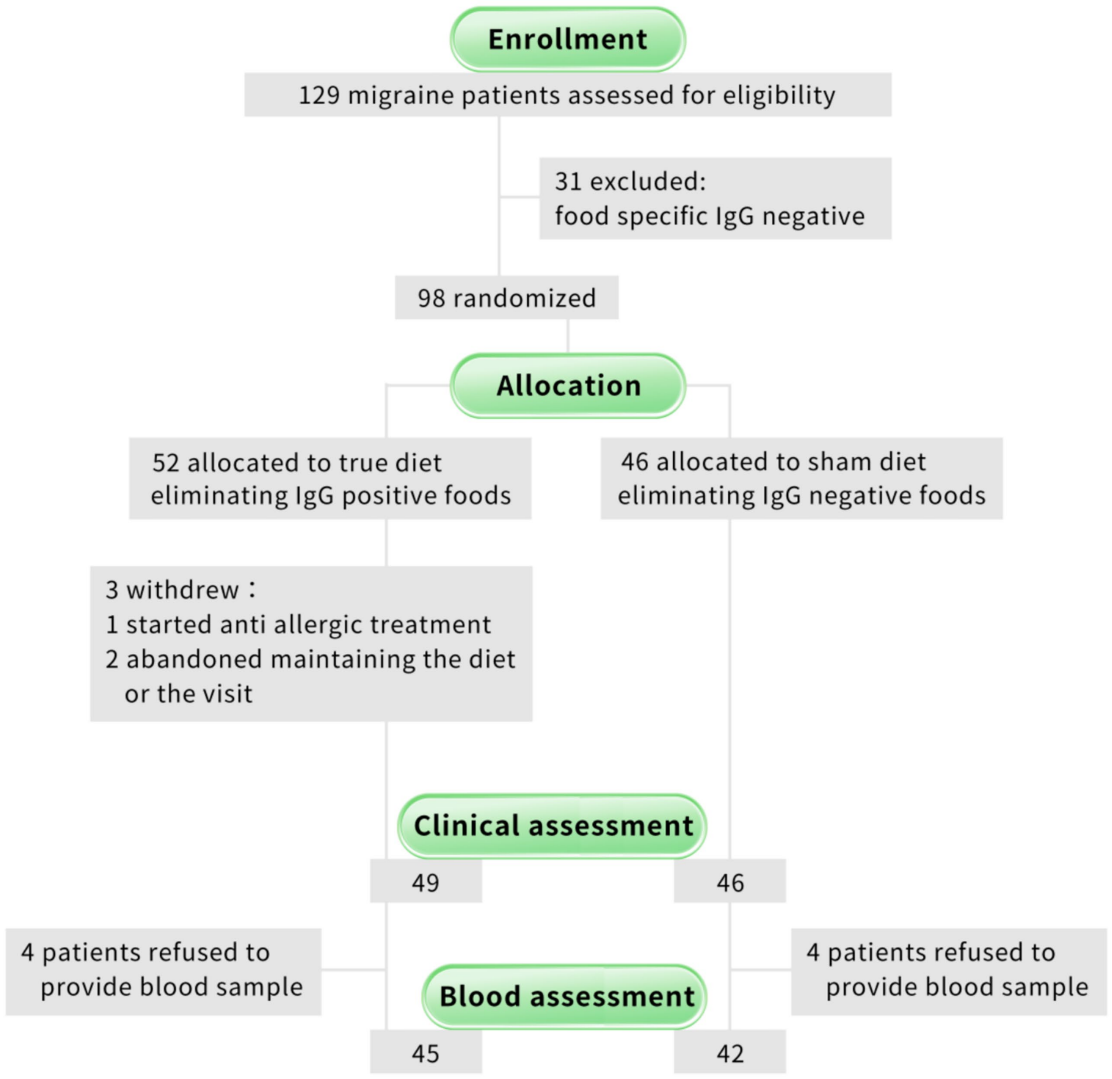
Study flow diagram.

This was a single-blinded sham-controlled randomized trial (Figure 1). The serum concentration of 14 different types of food-specific IgG was tested at baseline. Migraineurs who had no positive food-specific IgG were excluded and informed of their true test results. The subjects who had at least one positive food-specific IgG were randomly assigned to the “true” or “sham” dietary group by randomly selecting opaque envelopes coded by an independent staff member, who developed a personalized elimination diet sheet and follow-up schedule for the subjects. The subjects were asked to keep a headache diet diary and exclude designated foods for 12 weeks while maintaining their other original diet and lifestyle habits without intentionally eliminating other foods. During the study, all patients, laboratory staff, and statisticians were unaware of the randomized allocation plan until the completion of the study. Communication related to the efficacy effect was restricted between participants and study personnel to reduce the risk of unblinding and to maintain the integrity of the study.

### 2.3 Dietary intervention

The intervention for the “true” group was defined as eliminating IgG-positive foods; the intervention for the “sham” group was defined as eliminating alternative foods but not truly positive foods.

Alternative foods in the sham diet were IgG-negative and selected from the list of 14 tested foods. Table 1 shows the alternative food sheet of the sham diet. We swapped and matched 14 types of foods based on animal/plant foods, and the difficulty was eliminated according to the dietary habits of the local residents. On one hand, we could ensure that IgG-negative foods were indeed excluded from the sham diet, avoiding allergenic foods other than the 14 types of foods. On the other hand, blindness will not be broken when patients learn that their fasting foods are indeed tested foods, thereby improving compliance.

**Table 1:** Alternative food sheet of the sham elimination diet.

Positive food	Alternative food^*^
Animal origin food	
1 Eggs	1 Cow’s Milk
2 Cow’s Milk	2 Eggs
3 Pork	3 Chicken
4 Chicken	4 Pork
5 Beef	5 Shrimp
6 Shrimp	6 Beef
7 Codfish	7 Crab
8 Crab	8 Codfish
Plant origin food	
1 Rice	1 Wheat
2 Wheat	2 Rice
3 Soybean	3 Corn
4 Corn	4 Soybean
5 Tomato	5 Mushroom
6 Mushroom	6 Tomato

^*^If two or more foods are excluded at the same time, the matching order will be postponed accordingly.

Dietary compliance rate = number of meals in compliance with the dietary plan/total number of meals × 100% (25). We encourage patients to have three meals every day, resulting in a total of 252 meals for 12 weeks. A dietician was consulted to confirm that the alternative diet plan is nutrient-adequate and the two diets provide roughly the same overall calorie and macronutrient distribution.

### 2.4 Clinical assessment

At baseline, the demographic information and past medical history related to migraine were gathered, including age, gender, BMI (kg/m^2^), aura, episodic/chronic migraine, and the length of diagnosis.

Seven questionnaires were administered to measure the migraine-related disability, impact on quality of life, severity, and the comorbidities at baseline and the 12-week endpoint: (1) migraine disability assessment (MIDAS) including visual analog scale (VAS), which was the primary endpoint; (2) migraine-specific quality of life questionnaire version 2.1 (MSQ), by raw dimension scores; (3) headache impact test-6 (HIT-6); (4) gastrointestinal symptom rating scale (GSRS); (5) self-rating anxiety scale (SAS); (6) self-rating depression scale (SDS); (7) Pittsburgh sleep quality index (PSQI).

### 2.5 Blood variables analysis

Fasting blood was collected at baseline and the 12-week endpoint. All the enzyme-linked immunosorbent assays (ELISA) were performed using Biochrom Anthos 2010 microplate reader (Biochrom Ltd., Cambourne, Cambridge, UK) at 450 nm, according to the manufacturer’s recommendations. The procedure to quantify the levels was followed based on the manufacturer’s standard guidelines.

The serum concentration of 14 different types of food-specific IgG was determined by the ELISA kits (Bioeurope GmbH, Germany). Food-specific IgG ≥ 50 U/mL was defined as positive. All the food-specific positive IgG concentrations were summed up to derive a total positive IgG.

Corresponding ELISA kits were used to measure the serum concentration of IL-6, TNF-α, CGRP, 5-HT (BIM, San Francisco, USA), IL-10, and VIP (HengYuan Biological Technology Co., Ltd., Shanghai, China).

### 2.6 Ethical consideration

Our study was conducted according to the guidelines of the Declaration of Helsinki and approved by the Ethics Committee of the First Affiliated Hospital of Harbin Medical University (Protocol code 2019XS38-02, December 13, 2019). Written informed consent was obtained from the study participants. We registered our research in the Chinese Clinical Trial Registry (Chictr.org.cn, identifier ChiCTR2000039278, October 22, 2020).

### 2.7 Statistical analysis

Demographic and baseline characteristics were summarized using descriptive statistics for the two diet groups. The mean with standard deviation and the two-sample t-test was used to describe continuous endpoints and to test the baseline difference between two groups. For the categorical endpoints, the count with proportion and the chi-squared test were performed.

For both the sham diet group and the true diet group, the migraine and morbidity endpoints were reported at baseline and at week 12. The changes from baseline were assessed using a paired *t*-test for the continuous endpoints (i.e., MIDAS, HIT-6, etc.) and McNemar’s test for the binary endpoints (i.e., depression, anxiety, etc.). To assess the difference in the change of continuous endpoints from baseline between the two diet groups, linear regression models were performed using the diet group as the factor variable and baseline value as the covariate. For the binary endpoints, generalized linear regression models were performed by using visit and diet group as the factor variables, the respective baseline continuous variables as the covariates (i.e., SAS, SDS, etc.), along with the interaction of visit and diet group.

For the subjects with complete blood assessment at baseline and at week 12, the changes over 12 weeks were calculated for each diet group, and a paired *t*-test was used to assess the change over time within each diet group. Two-sample independent t-tests were used to test the difference in the change between the two diet groups.

Mixed models for repeated measures (MMRM) were performed to assess the 12-week change of continuous endpoints of migraine and comorbidities between the two diet groups. These models used visit and diet group, and the factor variables; baseline assessment as the covariate; and the interaction between visit and diet group.

Logistic regression mode was performed to test if the two diet groups were significantly different in the change of the IgG-positive rate from baseline. The butterfly graph was used to display the frequency of each positive IgG at baseline and week 12 for the two diet groups.

To assess the effect of inflammatory factors, the models were performed with IL-6 and TNT-*α*, unadjusted and adjusted. The unadjusted and adjusted differences in the change between the two groups and 95% CIs were reported.

All the statistical analyses were performed using Statistical Analysis Software (version 9.4; SAS Institute, Inc., Cary, NC, USA).

## 3 Results

### 3.1 Patients

From October 28, 2020, to March 11, 2021, a total of 129 migraineurs who met the clinical inclusion criteria were recruited. After blood tests, 98 patients had at least one specific IgG antibody-positive food. A total of 52 were randomly assigned to the true diet group, and 46 were randomized to the sham diet group. Characteristics of patients did not show a significant difference between the two groups at baseline (Table 2). A total of 31 migraineurs (24%) with no positive IgG tests were excluded (Figure 1).

**Table 2:** Baseline demographic and characteristics of the two groups.

Characteristic^a^	Sham diet (*N* = 46)	True diet (*N* = 49)	*p*-value^b^
Age at enrollment	40.5 (7.84)	38.2 (8.88)	0.1886
Male *n* (%)	15 (32.6)	16 (32.7)	0.9963
BMI (kg/m^2^)	24.2 (3.46)	23.5 (3.88)	0.3786
With aura, *n* (%)	3 (6.5)	9 (18.4)	0.0824
Chronic migraine, *n* (%)	4 (8.7)	5 (10.2)	0.8019
Time since diagnosis at enrollment (month)	90.6 (69.81)	112.1 (101.09)	0.2293

a Continuous endpoints were summarized using mean (SD); categorical endpoints were summarized using count (%).

b *p*-value was from an independent two-group *t*-test for continuous endpoints and chi-squared test for categorical endpoints.

At the endpoint of 12 weeks, 95 patients completed the post-intervention questionnaire, among whom 87 patients completed blood tests. Figure 1 shows the flow chart of the study and the number of patients throughout the study.

### 3.2 Compliance and adverse events

According to a similar research experience, a compliance rate ≥ 70% is considered as compliance with dietary intervention plans (17, 26), and all the patients have met this standard in our study. Eighty-three patients (87%) reported following their diet plan for 12 weeks (76–100% of the time), and the other 12 patients (13%) reported frequently following their diet plan (70–75% of the time) (27). There was no statistically significant difference in dietary compliance(%) between the two groups at 12 weeks (84.2 ± 8.2 vs. 86.2 ± 9.8, *p* < 0.01). No serious adverse events were reported.

### 3.3 Clinical endpoints

The clinical symptoms of migraines and comorbidities in 95 patients who completed the post-intervention questionnaire were analyzed. Comparison of the clinical endpoints between the two dietary groups is seen in Table 3. The true diet group had a larger reduction in migraine (except for MSQ), gastrointestinal, and sleep symptoms compared to the sham diet group. However, there was no significant difference in the reduction of MSQ, SDS, SAS, anxiety, depression, chronic migraine, and BMI between the two groups.

**Table 3:** Evaluation of clinical characteristics of migraine and its comorbidities before and after the intervention.

Characteristic	Sham diet (*N* = 46)^a^				True diet (*N* = 49)^a^				*p* value^b^
	Baseline	Week 12	Change (95% CI)	*p* value^a^	Baseline	Week 12	Change (95% CI)	*p* value^a^	
MIDAS	44.0 (27.08)	41.3 (27.92)	−2.6 (−4.4, −0.9)	0.0044	49.2 (35.80)	38.9 (36.11)	−10.3 (−15.3, −5.4)	0.0001	0.0074
N of days with migraine in past12 weeks	17.4 (13.58)	16.5 (12.82)	−0.9 (−1.7, −0.1)	0.0255	20.4 (19.67)	16.1 (17.93)	−4.2 (−6.2, −2.3)	<0.0001	0.0040
VAS	6.6 (1.24)	5.8 (1.35)	−0.8 (−1.2, −0.5)	<0.0001	7.0 (1.41)	4.9 (1.76)	−2.1(−2.5, −1.6)	<0.0001	0.0002
HIT - 6	59.0 (4.37)	58.1 (5.09)	−1.0 (−2.3, 0.4)	0.1567	62.3 (6.47)	56.2 (8.09)	−6.1 (−8.1, −4.1)	<0.0001	0.0013
N of days with migraine in past 4 weeks	5.8 (4.39)	5.4 (4.35)	−0.4 (−0.8, 0)	0.0527	6.7 (6.48)	5.3 (5.71)	−1.4 (−2.2, −0.7)	<0.0001	0.0283
MSQ	28.2 (9.38)	25.7 (7.58)	−2.5 (−4.4, −0.6)	0.0119	33.7 (14.39)	26.8 (11.65)	−6.9 (−9.9, −4.0)	<0.0001	0.1529
GSRS	27.6 (7.20)	27.4 (6.47)	−0.2 (−1.3, 1.0)	0.7582	33.2 (12.29)	26.3 (9.57)	−6.9 (−9.7, −4.2)	<0.0001	0.0011
SDS	40.9 (8.52)	38.8 (7.39)	−2.1 (−4.0, −0.3)	0.0259	45.6 (12.20)	41.3 (11.57)	−4.3 (−7.7, −0.9)	0.0151	0.9436
Depression, n (%)	9 (19.6)	5 (10.9)	−8.7 (−20.5, 3.1)	0.1573	17 (34.7)	11 (22.4)	−12.2 (−25.7, 1.2)	0.0833	0.6970
SAS	40.8 (7.80)	37.8 (7.95)	−2.9 (−5.0, −0.8)	0.0078	44.0 (11.39)	38.6 (10.52)	−5.4 (−8.4, −2.5)	0.0006	0.5228
Anxiety, *n* (%)	8 (17.4)	5 (10.9)	−6.5 (−19.2, 6.1)	0.3173	13 (26.5)	9 (18.4)	−8.2 (−21.8, 5.5)	0.2482	0.8819
PSQI	7.3 (3.26)	7.0 (3.05)	−0.3 (−1.0, 0.4)	0.3837	8.4 (3.65)	6.1 (3.60)	−2.3 (−3.3, −1.3)	<0.0001	0.0069
Poor sleeping, *n* (%)	19 (41.3)	18 (39.1)	−2.2 (−13.4, 9.1)	0.7055	24 (49.0)	13 (26.5)	−22.5 (−34.1, −10.8)	0.0009	0.0156
Chronic migraine, *n* (%)	4 (8.7)	4 (8.7)	0	–	5 (10.2)	3 (6.1)	−4.1 (−9.6, 1.5)	0.1573	0.1540
BMI (kg/m^2^)	24.0 (3.3)	23.9 (3.3)	−0.03 (−0.2, 0.11)	0.65	23.7 (3.8)	23.5 (4.1)	−0.2 (−0.8, 0.3)	0.33	0.42

a Change of characteristics since baseline, 95% CI, and *p*-value within each diet group are calculated from a paired *t*-test for continuous endpoints and McNemar’s test for categorical endpoints.

b For the comparison of change between diet groups for continuous endpoints, the *p*-value is derived from a linear regression model with diet group as a factor and baseline value as a covariate. For the categorical endpoints (except for the endpoint of chronic migraine), a *p*-value is derived from generalized linear regression models by using visit and diet group as factor variables, along with the interaction of visit and diet, and baseline measures as covariates. For the endpoint of chronic migraine, no baseline covariate was used.

What is more, the reduction of MIDAS and VAS after true dietary intervention also reached the minimal clinically important difference(MCID) as the primary outcome. The reduction of the MIDAS score was not only significantly higher than that in the sham diet group (10.3 vs. 2.6) but also higher than the minimal important change (MIC) (28). The reduction rate (%) of MIDAS in the true diet group was also higher than that in the sham diet group (29.7% vs. 8.8%), and closer to 30%, which was the minimal important difference(MID) (29). VAS in the true diet group was significantly higher than that in the sham diet group (2.1 vs. 0.8) and reached MCID (30).

### 3.4 Food-specific IgG antibody

Since the clinical symptom scales showed that dietary restriction therapy can improve some symptoms of migraine and its comorbidities, we investigated whether the two diets could regulate the levels of food-specific IgG in the serum of migraine patients. We selected 87 patients who completed post-intervention blood collection, which included 45 patients in the true diet group and 42 patients in the sham diet group. At the end of week 12, the total positive IgG concentration, IgG-positive rate, and number of IgG-positive foods were significantly reduced in the true diet group compared to baseline (Table 4). Comparing the two dietary groups, the reduction in the three variables was significantly greater in the true dietary group than in the sham dietary group.

**Table 4:** Comparison of effects on food specific IgG between two groups.

Variables	“Sham diet” (*n* = 42)^a^				“True diet” (*n* = 45)^a^				*p* value^b^
	Baseline	Week 12	Change (95% CI)	*p* value	Baseline	Week 12	Change (95% CI)	*p* value	
Total positive IgG concentration (u/mL)	213.9 (125.4)	179.6 (125.6)	−34.3 (−56.1, −12.5)	0.0028	223.6 (176.4)	78.1 (98.8)	−145.5 (−187.0, −104.1)	<0.0001	<0.0001
IgG positive rate (%)	100	88.1	−11.9 (−21.7, −2.1)	0.0625	100	64.4	−35.6 (−49.5, −21.6)	<0.0001	<0.0135
N of IgG positive foods	1.6 (0.6)	1.5 (0.9)	−0.1 (−0.4, 0.1)	0.4003	1.7 (0.8)	0.8 (0.7)	−0.9 (−1.2–0.7)	<0.0001	<0.0001

a Change of variables since baseline, 95% CI, and *p*-value within each diet group are calculated from a paired *t*-test for continuous endpoints and McNemar’s test for categorical endpoints.

b For the comparison of changes in total IgG concentration and frequency of IgG-positive food between diet groups, *p*-values were derived from linear regression models with diet group as a factor and baseline value as a covariate. For comparing the change in IgG-positive rate between two groups, and *p*-value was derived from a logistic regression model by using diet group as the factor variable.

**Figure 2:**
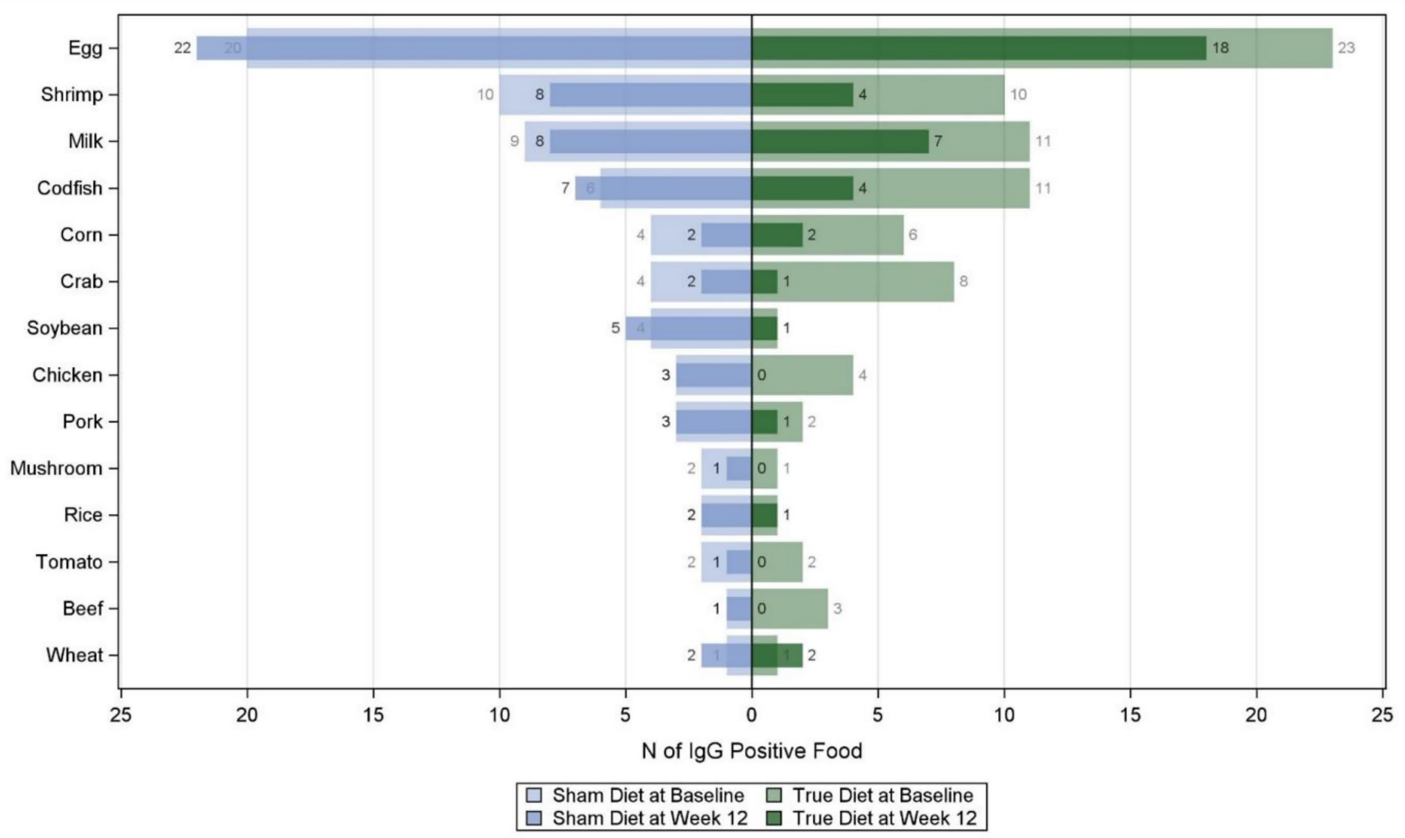
Comparison of the number of IgG-positive foods between the two groups at baseline and week 12.

We compared the changes in the number of positive cases of each food before and after 12 weeks between the two diets. As shown in Figure 2, there were 11 foods in the true diet group that showed a decrease in the number of positive cases: codfish and crab (7), shrimp (6), egg (5), milk, chicken, and corn (4), beef (3), tomato (2), pork and mushroom (1). In the sham diet group, the number of positive cases decreased for six foods: shrimp, crab, corn (2), milk, tomato, and mushroom (1).

### 3.5 Comparison of two diets on the levels of IL-6, IL-10, TNF-α, CGRP, 5-HT, and VIP

The concentrations of IL-6, TNF- α, and CGRP significantly decreased in the true diet group compared with both baseline and the sham diet group. However, although IL-10 and 5-HT increased in the true diet group and decreased in the sham diet group, the changes were not significant compared with either baseline or the other group. There were no significant changes in VIP (Table 5).

**Table 5:** Comparison of two diets on the levels of serum variables.

Variable	“Sham diet” (*N* = 42)				“True diet” (*N* = 45)				*p* value^b^
	Baseline	Week 12	Change (95% CI)	*p* value^a^	Baseline	Week 12	Change (95% CI)	*p* value^a^	
IL-6 (pg/mL)	13.5 (5.89)	13.8 (6.06)	0.4 (−1.0, 1.8)	0.5763	13.0 (7.67)	9.2 (3.81)	−3.8 (−5.7, −1.9)	0.0002	0.0005
IL-10 (ng/L)	128.8 (96.90)	123.7 (123.63)	−5.1 (−39.3, 29.1)	0.7650	122.5 (101.44)	136.5 (114.83)	14.0 (−21.4, 49.4)	0.4303	0.4364
TNF-α (pg/mL)	18.7 (6.69)	16.6 (7.95)	−2.1 (−3.6, −0.6)	0.0064	21.1 (14.18)	9.7 (6.96)	−11.4 (−15.0, −7.8)	<0.0001	<0.0001
CGRP (pg/mL)	33.7 (26.56)	32.4 (22.14)	−1.3 (−5.7, 3.2)	0.5594	27.8 (12.31)	18.3 (7.90)	−9.6 (−13.0, −6.2)	<0.0001	0.0039
5-HT (ng/mL)	198.2 (102.03)	188.8 (151.40)	−9.4 (−56.3, 37.5)	0.6870	202.9 (153.68)	231.8 (223.46)	28.9 (−39.7, 97.5)	0.4008	0.3555
VIP (ng/L)	54.2 (41.05)	67.2 (64.80)	13.0 (−8.7, 34.8)	0.2333	84.4 (71.67)	79.3 (67.98)	−5.1 (−29.4, 19.2)	0.6738	0.2649

a *p*-values were reported for the paired *t*-test used to compare the baseline and week 12 measures.

b *p*-values were reported from the two independent sample *t*-test used to compare the two groups in the change between baseline and week 12 measures.

### 3.6 The association between migraine and comorbidities and two diets before and after adjusting for the inflammatory cytokines

The result of mixed models for repeated measures (MMRM) showed greater change of migraine and comorbidity endpoints for the true diet group compared to the sham diet group. For example, the MIDAS score decreased by 2.7 (95% CI: −6.8 to 1.4) for the sham diet group and 11.4 (95% CI: −15.4, −7.4) for the true diet group at week 12. This change from the baseline between the two groups was significant (difference = −8.7, 95% CI: −14.5, −3.0, *p*-value: 0.0033). The highly significant differences were also observed for days with migraine in the past 12 and 4 weeks, VAS, MSQ, GSRS, and PSQI (i.e., *p*-value < 0.01). The differences were not statistically significant for SDS and SAS (i.e., *p*-values were 0.4759 and 0.263, respectively). Similar results of the difference in change from baseline were observed from both MMRM models with and without inflammatory factors adjusted. For days with migraine in the past 4 weeks, the difference in the change from baseline between the sham diet and the true diet groups was significantly attenuated after adjusting IL-6 and TNF-α (i.e., *p*-value = 0.0098 for the unadjusted model vs. *p*-value = 0.0757 for the adjusted model). As for other endpoints, except MSQ, the change from baseline between the sham diet and true diet groups was slight, and the largest attenuation was observed for the MIDAS (i.e., −8.7 for the unadjusted model vs. −7.1 for the adjusted model) (see Table 6).

**Table 6:** The association between Variables and two diets before and after adjusting for IL-6 and TNF-α.

Variable	Not adjusting for IL-6 and TNF-α				Adjusting for IL-6 and TNF-α			
	Change in sham diet over 12 weeks	Change in true diet over 12 weeks	Difference	*p* value	Change in sham diet over 12 weeks	Change in true diet over 12 weeks	Difference	*p* value
Migraine questionnaires								
MIDAS	−2.7 (−6.8, 1.4)	−11.4 (−15.4, −7.4)	−8.7 (−14.5, −3.0)	0.0033	−2.1 (−6.2, 2.0)	−9.2 (−13.6, −4.8)	−7.1 (−12.9, −1.2)	0.0182
VAS	−0.8 (−1.2, −0.4)	−2.2 (−2.6, −1.8)	−1.4 (−2.0, −0.9)	<0.0001	−0.8 (−1.2, −0.3)	−2.1 (−2.6, −1.7)	−1.4 (−2.0, −0.8)	<0.0001
HIT-6	−0.7 (−2.4, 1.1)	−6.8 (−8.5, −5.1)	−6.1 (−8.6, −3.7)	<0.0001	−0.6 (−2.4, 1.1)	−6.7 (−8.6, −4.9)	−6.1 (−8.6, −3.6)	<0.0001
MSQ	−2.3 (−4.9, 0.4)	−7.6 (−10.2, −5.0)	−5.3 (−9.0, −1.6)	0.0054	−2.2 (−4.9, 0.5)	−7.6 (−10.4, −4.9)	−5.4 (−9.2, −1.6)	0.0056
Days with Migraine in past 12 weeks	−1.0 (−2.7, 0.6)	−4.7 (−6.3, −3.1)	−3.6 (−5.9, −1.3)	0.0023	−0.9 (−2.5, 0.8)	−3.9 (−5.6, −2.2)	−3.0 (−5.4, −0.7)	0.0113
Days with Migraine in past 4 weeks	−0.4 (−1.0, 0.3)	−1.6 (−2.2, −1.0)	−1.2 (−2.1, −0.3)	0.0098	−0.3 (−0.9, 0.4)	−1.1 (−1.8, −0.4)	−0.8 (−1.8, 0.1)	0.0757
Comorbidities questionnaires								
GSRS	−0.3 (−2.6, 1.9)	−6.8 (−8.9, −4.6)	−6.4 (−9.6, −3.3)	<0.0001	−0.3 (−2.6, 2.0)	−6.4 (−8.8, −4.1)	−6.2 (−9.4, −3.0)	0.0003
SDS	−1.7 (−4.4, 1.0)	−3.1 (−5.7, −0.5)	−1.4 (−5.1, 2.4)	0.4759	−1.5 (−4.2, 1.2)	−2.5 (−5.3, 0.4)	−1.0 (−4.8, 2.9)	0.6185
SAS	−2.6 (−5.3, 0.1)	−4.8 (−7.4, −2.1)	−2.1 (−5.9, 1.6)	0.2634	−2.4 (−5.1, 0.4)	−4.0 (−6.8, −1.1)	−1.6 (−5.5, 2.3)	0.4159
PSQI	0 (−1.0, 0.9)	−2.4 (−3.2, −1.5)	−2.3 (−3.6, −1.1)	0.0004	0 (−0.9, 0.9)	−2.0 (−3.0, −1.1)	−2.1 (−3.4, −0.7)	0.0024
Blood variables								
5-HT	−9.4 (−69.2, 50.3)	28.9 (−28.8, 86.6)	38.3 (−44.8, 121.4)	0.3618	−5.2 (−64.8, 54.4)	46.1 (−16.2, 108.4)	51.3 (−33.5, 136.2)	0.2323
CGRP	−1.3 (−5.2, 2.6)	−9.6 (−13.4, −5.8)	−8.3 (−13.7,-2.8)	0.0035	−1.4 (−5.4, 2.5)	−8.3 (−12.3, −4.2)	−6.8 (−12.4, −1.3)	0.0171
VIP	13.0 (−10.2, 36.3)	−5.1 (−27.6, 17.3)	−18.2 (−50.4, 14.2)	0.2673	15.0 (−8.2, 38.3)	2.3 (−21.8, 26.4)	−12.8 (−45.8, 20.2)	0.4434

The changes over 12 weeks and the difference of the change between the two diet groups were reported from the MMRM models with diet group and visit as the factor variables, interaction between diet group and visit, and the baseline assessment as the covariates. The adjusted models included the same predictor variables as the unadjusted model, and IL-6 and TNF-α as the additional covariates.

## 4 Discussion

As we know, this is the first study to explore the effects of food food-specific IgG-based elimination diet on migraine and its comorbidities. According to our findings, the true diet, which excluded IgG-positive foods, significantly improved migraine (except for MSQ) and gastrointestinal and poor sleeping symptoms compared to the sham diet group.

As a cost-effective alternative, utilizing IgG food sensitivity testing to create customizable dietary recommendations for patients may reduce their dependence on medication (12). Therefore, researchers have been paying attention to the elimination diet for migraine. Alpay et al. conducted a 6-week dietary intervention that excluded IgG-positive foods and found that the number of headache days and attacks significantly decreased compared to the baseline phase (21). A clinical study on patients with migraine and irritable bowel syndrome (IBS) showed that exclusion of foods with positive IgG has effectively alleviated symptoms of migraine and irritable bowel syndrome (IBS) (22).

In addition, there are some elimination diet studies on gastrointestinal and sleep disorders. Singh et al. found that subjects with IBS on an IgG-guided elimination diet were more likely to meet the primary outcome (a ≥ 30% decrease in abdominal pain intensity) (31). A randomized control trial found that the subjects who avoided foods with high titers of IgG reported significant improvement in sleeping quality compared with the waitlist (23).

Our findings are consistent with the results from the previous studies, although there are more highlights. First, our data further confirmed the effectiveness of the IgG elimination diet not only on migraine but also on its comorbidities. This prompts us to propose a hypothesis that these comorbidity symptoms were not randomly occurring; they may have a common pathogenesis with migraine. Second, in order to explore the mechanisms behind the improvement of the above clinical symptoms, we investigated the effect of dietary intervention on the number of IgG-positive foods, total concentration of positive IgG antibodies, inflammatory cytokines, neuropeptides, and neurotransmitters, as well as their interaction.

As a result, it was found that a 12-week IgG-positive food elimination diet significantly reduced the total concentration of positive IgG antibodies, IgG-positive rate, and the number of IgG-positive foods. Among 14 types of food, animal foods were more likely to trigger sensitization, and the elimination diet with animal food excluded (especially seafood) was more likely to reduce the IgG level. Some scholars have proposed that animal foods (such as seafoods, red meat, eggs, and dairy products) have greater inflammatory potential than vegetables and fruits (24, 32, 33).

Ligaarden et al. (34) found that the level of food-specific IgG is directly proportional to the intake of those foods, which can be used to evaluate individual dietary habits and food antigen exposure, and to adopt personalized nutritional methods to attenuate this exposure and to reduce systemic inflammation. Our previous randomized controlled study on 214 IBS patients found that the 12-week IgG-positive elimination diet improved IBS symptoms, and the total positive IgG titer grade was significantly decreased from baseline (35), which is consistent with the results of this study. However, a study on overweight/obese adults found that the median percentage of foods with a class reduction was higher in the intervention group (85.7%) compared with the waitlist group (60%), but this comparison was likewise non-significant (23). The reasons may be that, on the one hand, the researchers only eliminated foods that had the highest concentration of IgG reaction (class 2 or 3) rather than all positive foods; on the other hand, they did not directly compare the IgG concentration of positive foods; instead, they compared the median percentage of foods with a class reduction. Therefore, we hypothesized that completely abstaining from all IgG-positive foods could be more effective than eliminating foods with the highest IgG antibody concentration, and the reduction of IgG concentration of positive foods may be a better indicator of the therapeutic effect of the elimination diet.

Our study found a significant decrease in concentrations of IL-6, TNF-α, and CGRP in the true diet group from baseline compared to the sham diet group. Furthermore, the results from the MMRM models indicated the potential effect of inflammatory factors as possible mediators under the mechanism that underlies the relationship between the IgG elimination diet, migraine, and comorbidity symptoms. After adjusting for IL-6 and TNF-α in the exploratory analysis, the positive effect of diet intervention on the severity of migraine and its comorbidities (except MSQ) was reduced, which supports our hypothesis that IL-6 and TNF-α partially mediated the causal pathway.

Food-specific IgG antibody can be implicated in systemic anaphylaxis (10, 36). Although complexes of food antigens bound to specific IgG circulating in the serum are the body’s regular reactions to food antigens. The antibody production depends on exposure and the immunogenicity of the foods (37). Excessive food-specific IgG antibodies can bind to food antigens passing through the intestinal mucosa to form immune complexes, which are deposited in the blood vessels. They may also play a role in the pathogenesis of chronic intestinal inflammations by contributing to increased cuticular permeability (38). The presence of these complexes activates B-cell responses, releases pro-inflammatory cytokines, including TNF-α and IL-6, and finally causes low-grade chronic inflammation throughout the body (39, 40). TNF-α and IL-6 are classic pro-inflammatory cytokines that have been widely investigated in many migraine studies (41, 42). Studies have shown that cytokines and CGRP may jointly participate in the pathogenesis of migraine. Inflammatory cytokines can activate nociceptors in the central and peripheral nervous systems, and the release of CGRP from the trigeminal nerve can stimulate inflammation and immune responses, leading to migraine attacks (43, 44). In addition, comorbidities of digestion, sleep, anxiety, and depression are also manifestations of this systemic inflammation (40, 45–48).

We speculate accordingly that relieved migraine, sleep, and gastrointestinal symptoms observed in our study may be due to the reduction of food-specific IgG antibody production by elimination diet, which in turn reduced the release of IL-6 and TNF-α (serum concentration decreases) and alleviated systemic inflammation. Subsequently, downregulation of the sensitivity of trigeminal nerve endings to nociceptors reduces the release and activity of CGRP, leading to the alleviation of migraine symptoms. Meanwhile, the reduction of migraine attacks may also lead to a decrease in CGRP release. The latest research has found that anti-inflammatory interventions not only alleviate pain hypersensitivity in mice with inflammatory pain but also reduce peripheral blood CGRP levels (49), which supports the results of our study.

Our study emphasizes the fact that excluding food-specific IgG-positive foods for 12 weeks did not significantly alter the random serum levels of 5-HT, VIP, and IL-10. The purpose of our study was to observe the effect of a 12-week daily dietary intervention on patients with migraine. Therefore, blood samples were collected at random times, and there was no special requirement for attacking or an intermittent period. Nevertheless, the concentration of 5-HT in the periphery and brain can be affected by the timing of migraine attacks. 5-HT levels increase during migraine attacks, causing vasoconstriction of cerebral arteries and triggering the prodromal phase and cerebral ischemia, leading to visual impairment. But as it is decomposed and excreted, the concentration of 5-HT will eventually decrease. After the concentration of 5-HT decreases to a certain extent, it can lead to the dilation of extracranial arteries, resulting in a new round of headache attacks (50, 51). Xie et al. found that a diet based on IgG elimination significantly reduced the number of migraine days and increased serum 5-HT levels at the end of the 14 weeks, with no difference at the end of the 7 weeks (52). Yet, our study has observed an increase in 5-HT levels at the end of the 12 weeks, although not statistically significant. Based on this, it is speculated that if the duration of dietary restriction continues to be extended after 12 weeks, there may also be statistically significant changes in serum 5-HT levels, which need to be confirmed through longer-term and more detailed subtype grouping comparisons.

The biological role of VIP in migraine is also extremely complex. On the one hand, VIP plays an effective role as an anti-inflammatory factor by inhibiting the production of pro-inflammatory cytokines but promoting anti-inflammatory cytokines (53, 54). This may explain why there was a certain degree of attenuation in the difference of VIP changes after adjusting for IL-6 and TNF-α. On the other hand, VIP may cause migraine attacks (8, 55) and inflammatory bowel disease (56) through vasodilation and degranulation of mast cells on the dura mater. It is speculated that VIP may mediate protective and adaptive responses, which aim to protect the nervous, mental, and gastrointestinal systems of patients with migraine from damage caused by pro-inflammatory factors and ensure the homeostasis of the brain–gut nervous system during IgG-mediated food allergy reactions. Therefore, serum concentration of VIP observed during the dietary intervention process in our study may also be adaptive changes. Further research can increase the observation time points to clarify whether there is a definite decrease or increase in VIP concentration during dietary intervention.

The compensatory increase of IL-10 is to suppress the sustained pro-inflammatory state, but the compensatory increase of IL-10 cannot completely offset the increase in IL-6 levels. Ultimately, the pro-inflammatory state dominates, whereas IL-10 is relatively insufficient (15). Oliveira et al. (57) also found that the TNF-α and IL-10 balance may be related to migraine pathomechanisms and its psychiatric comorbidities. We conclude that the latency between the stimulus of IL-10 and the reduction of IL-6 and TNF-α is perhaps not synchronized. Distinguishing between different timings of attack and long-term longitudinal studies are needed in the future to confirm this inference.

Despite efforts to improve our study, limitations still exist. First, our study was carried out during the COVID-19 epidemic in China. Such a sudden and significant public health crisis might have exacerbated anxiety, depression, and sleep disorders as well as headaches (58, 59), although there were no confirmed cases of COVID-19 infection among 98 subjects during the research period. The prevalence of anxiety and sleep disorders and the scores of patient-reported questionnaires at baseline were higher in our study compared to Zhao et al.’s (60) study with episodic migraine in China in 2014. After dietary intervention, there was no significant reduction in anxiety, depression, and MSQ between the two groups, which may be related to this influencing factor. Furthermore, the change of MSQ from baseline was not decreased after adjusting IL-6 and TNF-α, which may be because the MSQ is a 14-item disease-specific patient-reported outcome instrument, including three items assessing the emotions (61). Second, our study was conducted in a single region; the sample size and the observation period are relatively insufficient, which might compromise the power to detect a significant correlation. The observed correlation coefficient between inflammatory cytokines and clinical symptoms in this study is not high, and the lack of difference in IL-10, 5-HT, and VIP between the true and sham diet groups reflects insufficient strength. Therefore, a multicenter clinical trial with a bigger sample size and follow-ups of longer duration are needed to enhance the generalizability of our research findings. Subsequent similar studies should include unblinding testing to further enhance the reliability of the conclusions. Third, our study excluded patients with autoimmune diseases because of their impact on systemic chronic inflammation. However, it was found that patients with Hashimoto’s thyroiditis are more likely to have IgG-positive foods, and different dietary patterns may affect the activity of the thyroid axis and may even be the cause of autoimmune disease (62). Future research may consider the impact of an elimination diet on patients with migraine with autoimmune diseases and related mechanisms.

## 5 Conclusion

In summary, we provide clinical evidence that the IgG-positive food elimination diet was beneficial to migraine and its comorbidities and reduced the production of IL-6, TNF-α, and CGRP. This therapeutic effect might be associated with the alleviated systemic chronic inflammation and downregulation of the sensitivity of trigeminal nerve endings to nociceptors. Future work should explore potential mechanisms, such as the role of gut microbiota and intestinal permeability in the microbiota gut–brain axis.

## Data Availability

The raw data supporting the conclusions of this article will be made available by the authors, without undue reservation.

## References

[1] Burch RC Buse DC Lipton RB. Migraine: epidemiology, burden, and comorbidity. Neurol Clin. (2019) 37:631–49. doi: 10.1016/j.ncl.2019.06.001, 31563224

[2] Buse DC Reed ML Fanning KM Bostic R Dodick DW Schwedt TJ . Comorbid and co-occurring conditions in migraine and associated risk of increasing headache pain intensity and headache frequency: results of the migraine in America symptoms and treatment (MAST) study. J Headache Pain. (2020) 21:23. doi: 10.1186/s10194-020-1084-y, 32122324 PMC7053108

[3] Lu G Xiao S He J Xie W Ge W Meng F . Prevalence of depression and its correlation with anxiety, headache and sleep disorders among medical staff in the Hainan Province of China. Front Public Health. (2023) 11:1122626. doi: 10.3389/fpubh.2023.1122626, 37441641 PMC10333496

[4] Caponnetto V Deodato M Robotti M Koutsokera M Pozzilli V Galati C . Comorbidities of primary headache disorders: a literature review with meta-analysis. J Headache Pain. (2021) 22:71. doi: 10.1186/s10194-021-01281-z, 34261435 PMC8278743

[5] Raggi A Leonardi M Arruda M Caponnetto V Castaldo M Coppola G . Hallmarks of primary headache: part 1 - migraine. J Headache Pain. (2024) 25:189. doi: 10.1186/s10194-024-01889-x, 39482575 PMC11529271

[6] Minen MT Begasse De Dhaem O Kroon Van Diest A Powers S Schwedt TJ Lipton R . Migraine and its psychiatric comorbidities. J Neurol Neurosurg Psychiatry. (2016) 87:741–9. doi: 10.1136/jnnp-2015-312233, 26733600

[7] Nguyen KV Schytz HW. The evidence for diet as a treatment in migraine-a review. Nutrients. (2024) 16:3415. doi: 10.3390/nu16193415, 39408380 PMC11478386

[8] Spekker E Tanaka M Szabó Á Vécsei L. Neurogenic inflammation: the participant in migraine and recent advancements in translational research. Biomedicine. (2021) 10:76. doi: 10.3390/biomedicines10010076, 35052756 PMC8773152

[9] Dixon HS. Treatment of delayed food allergy based on specific immunoglobulin G RAST testing. Otolaryngol Head Neck Surg. (2000) 123:48–54. doi: 10.1067/mhn.2000.106402, 10889481

[10] Ostrowska L Wasiluk D Lieners CFJ Gałęcka M Bartnicka A Tveiten D. Igg food antibody guided elimination-rotation diet was more effective than FODMAP diet and control diet in the treatment of women with mixed IBS-results from an open label study. J Clin Med. (2021) 10:4317. doi: 10.3390/jcm10194317, 34640335 PMC8509634

[11] Demartini C Francavilla M Zanaboni AM Facchetti S De Icco R Martinelli D . Biomarkers of migraine: an integrated evaluation of preclinical and clinical findings. Int J Mol Sci. (2023) 24:5334. doi: 10.3390/ijms24065334, 36982428 PMC10049673

[12] Geiselman JF. The clinical use of IgG food sensitivity testing with migraine headache patients: a literature review. Curr Pain Headache Rep. (2019) 23:79. doi: 10.1007/s11916-019-0819-4, 31456119

[13] Sudershan A Sudershan S Sharma I Kumar H Panjaliya RK Kumar P. Role of TNF-α in the pathogenesis of migraine. Pain Res Manag. (2024) 2024:1–12. doi: 10.1155/2024/1377143, 38213956 PMC10781531

[14] Biscetti L De Vanna G Cresta E Corbelli I Gaetani L Cupini L . Headache and immunological/autoimmune disorders: a comprehensive review of available epidemiological evidence with insights on potential underlying mechanisms. J Neuroinflammation. (2021) 18:259. doi: 10.1186/s12974-021-02229-5, 34749743 PMC8573865

[15] Rong YD Bian AL Hu HY Ma Y Zhou XZ. Study on relationship between elderly sarcopenia and inflammatory cytokine IL-6, anti-inflammatory cytokine IL-10. BMC Geriatr. (2018) 18:308. doi: 10.1186/s12877-018-1007-9, 30541467 PMC6292155

[16] Stakenborg N Viola MF Boeckxstaens GE. Intestinal neuro-immune interactions: focus on macrophages, mast cells and innate lymphoid cells. Curr Opin Neurobiol. (2020) 62:68–75. doi: 10.1016/j.conb.2019.11.020, 31862627 PMC7294228

[17] Cappelletti M Tognon E Vona L Basello K Costanzi A Speciani MC . Food-specific serum IgG and symptom reduction with a personalized, unrestricted-calorie diet of six weeks in irritable bowel syndrome (IBS). Nutr Metab. (2020) 17:101. doi: 10.1186/s12986-020-00528-x, 33292297 PMC7708901

[18] Zhao ZM Yang MM Zhao XS Wan FJ Ning BL Zhang LM . The impact of food specific IgG antibodies on migraine and its comorbidities. Immun Inflamm Dis. (2024) 12:e70056. doi: 10.1002/iid3.70056, 39552191 PMC11570760

[19] Zhao Z Jin H Yin Y Hou Y Wang J Tang C . Association of Migraine with its comorbidities and food specific immunoglobulin G antibodies and inflammatory cytokines: cross-sectional clinical research. J Pain Res. (2021) 14:2359–68. doi: 10.2147/JPR.S316619, 34385841 PMC8352645

[20] Headache Classification Committee of the International Headache Society (IHS). The international classification of headache disorders, 3rd edition (beta version). Cephalalgia. (2013) 33:629–808. doi: 10.1177/0333102413485658, 23771276

[21] Alpay K Ertas M Orhan EK Ustay DK Lieners C Baykan B. Diet restriction in migraine, based on IgG against foods: a clinical double-blind, randomised, cross-over trial. Cephalalgia. (2010) 30:829–37. doi: 10.1177/0333102410361404, 20647174 PMC2899772

[22] Aydinlar EI Dikmen PY Tiftikci A Saruc M Aksu M Gunsoy HG . IgG-based elimination diet in migraine plus irritable bowel syndrome. Headache. (2013) 53:514–25. doi: 10.1111/j.1526-4610.2012.02296.x, 23216231

[23] Neuendorf R Corn J Hanes D Bradley R. Impact of food immunoglobulin G-based elimination diet on subsequent food immunoglobulin G and quality of life in overweight/obese adults. J Altern Complement Med. (2019) 25:241–8. doi: 10.1089/acm.2018.0310, 30265560

[24] Casini I Fatighenti E Giannantoni A Massai L Pieretti S Ceccarelli I . Food-specific IgG4 antibody-guided exclusion diet improves conditions of patients with chronic pain. Pain Ther. (2022) 11:873–906. doi: 10.1007/s40122-022-00391-z, 35612757 PMC9314524

[25] Mitchell N Hewitt CE Jayakody S Islam M Adamson J Watt I . Randomised controlled trial of food elimination diet based on IgG antibodies for the prevention of migraine like headaches. Nutr J. (2011) 10:85. doi: 10.1186/1475-2891-10-85, 21835022 PMC3199755

[26] Blannin A Boulton G Thielecke F. Six weeks of either EPA-rich or DHA-rich Omega-3 supplementation alters submaximal exercise physiology in endurance trained male amateurs. Front Nutr. (2025) 12:1588421. doi: 10.3389/fnut.2025.1588421, 40933262 PMC12417169

[27] Staudacher HM Lomer MCE Farquharson FM Louis P Fava F Franciosi E . A diet low in FODMAPs reduces symptoms in patients with irritable bowel syndrome and a probiotic restores Bifidobacterium species: a randomized controlled trial. Gastroenterology. (2017) 153:936–47. doi: 10.1053/j.gastro.2017.06.010, 28625832

[28] Carvalho GF Luedtke K Braun T. Minimal important change and responsiveness of the migraine disability assessment score (MIDAS) questionnaire. J Headache Pain. (2021) 22:126. doi: 10.1186/s10194-021-01339-y, 34674632 PMC8529733

[29] Ruscheweyh R Förderreuther S Freilinger T Gaul C Goßrau G Jürgens TP . Minimal important difference of the migraine disability assessment (MIDAS): longitudinal data from the DMKG headache registry. Cephalalgia. (2024) 44:3331024241261077. doi: 10.1177/03331024241261077, 39033424

[30] Muñoz-Leyva F El-Boghdadly K Chan V. Is the minimal clinically important difference (MCID) in acute pain a good measure of analgesic efficacy in regional anesthesia? Reg Anesth Pain Med. (2020) 45:1000–5. doi: 10.1136/rapm-2020-101670, 32900985

[31] Singh P Chey WD Takakura W Cash BD Lacy BE Quigley EMM . A novel, IBS-specific IgG ELISA-based elimination diet in irritable bowel syndrome: a randomized, sham-controlled trial. Gastroenterology. (2025) 168:1128–1136.e4. doi: 10.1053/j.gastro.2025.01.223, 39894284

[32] Lopata AL Kleine-Tebbe J Kamath SD. Allergens and molecular diagnostics of shellfish allergy: part 22 of the series molecular Allergology. Allergo J Int. (2016) 25:210–8. doi: 10.1007/s40629-016-0124-2, 28239537 PMC5306157

[33] Eussen SRBM Wielders S de Rooij WE Van Ampting MTJ Van Esch BCAM de Vries JHM . Dietary composition of adult eosinophilic esophagitis patients is related to disease severity. Immun Inflamm Dis. (2024) 12:e1206. doi: 10.1002/iid3.1206, 38456617 PMC10921897

[34] Ligaarden SC Lydersen S Farup PG. IgG and IgG4 antibodies in subjects with irritable bowel syndrome: a case control study in the general population. BMC Gastroenterol. (2012) 12:166. doi: 10.1186/1471-230X-12-166, 23170971 PMC3526446

[35] Zhao XS Shi LJ Ning BL Zhao ZM Li XX Zhu MH . Efficacy of diet restriction with or without probiotic for treatment of patients with IBS-D: phase I-II clinical trial. Immun Inflamm Dis. (2023) 11:e857. doi: 10.1002/iid3.857, 37249280 PMC10165954

[36] Piuri G Ferrazzi E Speciani AF. Blind analysis of food-related IgG identifies five possible nutritional clusters for the Italian population: future implications for pregnancy and lactation. Nutrients. (2019) 11:1096. doi: 10.3390/nu11051096, 31108900 PMC6566756

[37] Coucke F. Food intolerance in patients with manifest autoimmunity. Observational study. Autoimmun Rev. (2018) 17:1078–80. doi: 10.1016/j.autrev.2018.05.011, 30213697

[38] Gocki J Bartuzi Z. Role of immunoglobulin G antibodies in diagnosis of food allergy. Postepy Dermatol Alergol. (2016) 4:253–6. doi: 10.5114/ada.2016.61600, 27605894 PMC5004213

[39] Kang S Keener AB Jones SZ Benschop RJ Caro-Maldonado A Rathmell JC . IgG-immune complexes promote B cell memory by inducing BAFF. J Immunol. (2016) 196:196–206. doi: 10.4049/jimmunol.1402527, 26621863 PMC4684997

[40] Labrosse R Graham F Caubet JC. Non-IgE-mediated gastrointestinal food allergies in children: an update. Nutrients. (2020) 12:2086. doi: 10.3390/nu12072086, 32674427 PMC7400851

[41] Bougea A Spantideas N Galanis P Katsika P Boufidou F Voskou P . Salivary inflammatory markers in tension type headache and migraine: the SalHead cohort study. Neurol Sci. (2020) 41:877–84. doi: 10.1007/s10072-019-04151-4, 31823093

[42] Rozen T Swidan SZ. Elevation of CSF tumor necrosis factor alpha levels in new daily persistent headache and treatment refractory chronic migraine. Headache. (2007) 47:1050–5. doi: 10.1111/j.1526-4610.2006.00722.x, 17635596

[43] Vural S Albayrak L. Can calcitonin gene-related peptide (CGRP) and pentraxin-3 (PTX-3) be useful in diagnosing acute migraine attack? J Recept Signal Transduct Res. (2022) 42:562–6. doi: 10.1080/10799893.2022.2097264, 35895308

[44] Holzer P Holzer-Petsche U. Constipation caused by anti-calcitonin gene-related peptide migraine therapeutics explained by antagonism of calcitonin gene-related peptide's motor-stimulating and Prosecretory function in the intestine. Front Physiol. (2022) 12:820006. doi: 10.3389/fphys.2021.820006, 35087426 PMC8787053

[45] Rudzki L Pawlak D Pawlak K Waszkiewicz N Małus A Konarzewska B . Immune suppression of IgG response against dairy proteins in major depression. BMC Psychiatry. (2017) 17:268. doi: 10.1186/s12888-017-1431-y, 28738849 PMC5525306

[46] Lamers F Milaneschi Y Smit JH Schoevers RA Wittenberg G Penninx BWJH. Longitudinal association between depression and inflammatory markers: results from the Netherlands study of depression and anxiety. Biol Psychiatry. (2019) 85:829–37. doi: 10.1016/j.biopsych.2018.12.020, 30819515

[47] Gomes AP Soares ALG Menezes AMB Assunção MC Wehrmeister FC Howe LD . Adiposity, depression and anxiety: interrelationship and possible mediators. Rev Saude Publica. (2019) 53:103. doi: 10.11606/S1518-8787.2019053001119, 31800914 PMC6863175

[48] Mohammadi S Mayeli M Saghazadeh A Rezaei N. Cytokines in narcolepsy: a systematic review and meta-analysis. Cytokine. (2020) 131:155103. doi: 10.1016/j.cyto.2020.155103, 32315956

[49] Wang Z Yang L Xu L Liao J Lu P Jiang J. Central and peripheral mechanism of MOTS-c attenuates pain hypersensitivity in a mice model of inflammatory pain. Neurol Res. (2024) 46:165–77. doi: 10.1080/01616412.2023.2258584, 37899006

[50] Tuka B Nyári A Cseh EK Körtési T Veréb D Tömösi F . Clinical relevance of depressed kynurenine pathway in episodic migraine patients: potential prognostic markers in the peripheral plasma during the interictal period. J Headache Pain. (2021) 22:60. doi: 10.1186/s10194-021-01239-1, 34171996 PMC8229298

[51] Nan N Gong MX Wang Q Li MJ Xu R Ma Z . Wuzhuyu decoction relieves hyperalgesia by regulating central and peripheral 5-HT in chronic migraine model rats. Phytomedicine. (2022) 96:153905. doi: 10.1016/j.phymed.2021.153905, 35026523

[52] Xie Y Zhou G Xu Y He B Wang Y Ma R . Effects of diet based on IgG elimination combined with probiotics on migraine plus irritable bowel syndrome. Pain Res Manag. (2019) 2019:1–6. doi: 10.1155/2019/7890461, 31531150 PMC6721378

[53] Maiti AK Sharba S Navabi N Lindén SK. Colonic levels of vasoactive intestinal peptide decrease during infection and exogenous VIP protects epithelial mitochondria against the negative effects of IFNγ and TNFα induced during *Citrobacter rodentium* infection. PLoS One. (2018) 13:e0204567. doi: 10.1371/journal.pone.0204567, 30252907 PMC6155558

[54] Sigalet DL Wallace LE Holst JJ Martin GR Kaji T Tanaka H . Enteric neural pathways mediate the anti-inflammatory actions of glucagon-like peptide 2. Am J Physiol Gastrointest Liver Physiol. (2007) 293:G211–21. doi: 10.1152/ajpgi.00530.2006, 17395898

[55] Pellesi L Al-Karagholi MA Chaudhry BA Lopez CL Snellman J Hannibal J . Two-hour infusion of vasoactive intestinal polypeptide induces delayed headache and extracranial vasodilation in healthy volunteers. Cephalalgia. (2020) 40:1212–23. doi: 10.1177/0333102420937655, 32594760

[56] Rychlik A Gonkowski S Całka J Makowska K. Vasoactive intestinal polypeptide (VIP) in the intestinal mucosal nerve fibers in dogs with inflammatory bowel disease. Animals. (2020) 10:1759. doi: 10.3390/ani1010175, 32998326 PMC7599766

[57] Oliveira AB Bachi ALL Ribeiro RT Mello MT Tufik S Peres MFP. Unbalanced plasma TNF-α and IL-12/IL-10 profile in women with migraine is associated with psychological and physiological outcomes. J Neuroimmunol. (2017) 313:138–44. doi: 10.1016/j.jneuroim.2017.09.008 28950996

[58] Shen X Yan S Cao H Feng J Lei Z Zhang W . Current status and associated factors of depression and anxiety among the Chinese residents during the period of low transmission of COVID-19. Front Psychol. (2021) 12:700376. doi: 10.3389/fpsyg.2021.700376, 34646194 PMC8503548

[59] Huang Y Zhao N. Generalized anxiety disorder, depressive symptoms and sleep quality during COVID-19 outbreak in China: a web-based cross-sectional survey. Psychiatry Res. (2020) 288:112954. doi: 10.1016/j.psychres.2020.112954, 32325383 PMC7152913

[60] Zhao H Xiao Z Zhang L Ford J Zhong S Ye W . Real-world treatment patterns and outcomes among patients with episodic migraine in China: results from the Adelphi migraine disease specific programme™. J Pain Res. (2023) 16:357–71. doi: 10.2147/JPR.S371887, 36762367 PMC9904300

[61] Rendas-Baum R Bloudek LM Maglinte GA Varon SF. The psychometric properties of the migraine-specific quality of life questionnaire version 2.1 (MSQ) in chronic migraine patients. Qual Life Res. (2013) 22:1123–33. doi: 10.1007/s11136-012-0230-7, 22797868 PMC3664759

[62] Yan M Wu H Zhang K Gong P Wang Y Wei H. Analysis of the correlation between Hashimoto's thyroiditis and food intolerance. Front Nutr. (2024) 11:1452371. doi: 10.3389/fnut.2024.1452371, 39403393 PMC11471614

